# The nomogram based on the 6-lncRNA model can promote the prognosis prediction of patients with breast invasive carcinoma

**DOI:** 10.1038/s41598-021-00364-w

**Published:** 2021-10-21

**Authors:** Dankun Luo, Wenchao Yao, Qiang Wang, Qiu Yang, Xuxu Liu, Yang Yang, Weihui Zhang, Dongbo Xue, Biao Ma

**Affiliations:** 1 Department of General Surgery, The First Affiliated Hospital of Harbin Medical University, 23 You Zheng Street, Harbin, 150001 China; 2 Arnie Charbonneau Cancer Institute, Departments of Biochemistry and Molecular Biology and Oncology, University of Calgary, 3330 Hospital Dr. NW, Calgary, AB T2N 4N1 Canada

**Keywords:** Breast cancer, Cancer models, Tumour biomarkers, Bioinformatics

## Abstract

Long non-coding RNA (lncRNA) is a prognostic biomarker for many types of cancer. Here, we aimed to study the prognostic value of lncRNA in Breast Invasive Carcinoma (BRCA). We downloaded expression profiles from The Cancer Genome Atlas (TCGA) datasets. Subsequently, we screened the differentially expressed genes between normal tissues and tumor tissues. Univariate Cox, LASSO regression, and multivariate Cox regression analysis were used to construct a lncRNA prognostic model. Finally, a nomogram based on the lncRNAs model was developed, and weighted gene co-expression network analysis (WGCNA) was used to predict mRNAs related to the model, and to perform function and pathway enrichment. We constructed a 6-lncRNA prognostic model. Univariate and multivariate Cox regression analysis showed that the 6-lncRNA model could be used as an independent prognostic factor for BRCA patients. We developed a nomogram based on the lncRNAs model and age, and showed good performance in predicting the survival rates of BRCA patients. Also, functional pathway enrichment analysis showed that genes related to the model were enriched in cell cycle-related pathways. Tumor immune infiltration analysis showed that the types of immune cells and their expression levels in the high-risk group were significantly different from those in the low-risk group. In general, the 6-lncRNA prognostic model and nomogram could be used as a practical and reliable prognostic tool for invasive breast cancer.

## Introduction

Invasive breast cancer (BRCA) is a highly heterogeneous malignant tumor originating from the breast tissue. This cancer has become one of the major health threats faced by women worldwide, seriously affecting both life span and quality of life^1,2^. Up to one in twelve females in Britain between the ages of 1 and 85 years will develop breast cancer^3^. In addition, invasive breast cancer is the leading cause of cancer-related mortality in developing countries^3,4^. At present, the prognosis and therapeutic strategy for breast cancer patients mainly depend on the TNM stage of the disease, histological grade, and the expression level of hormone receptors and human epidermal growth factor receptor 2 receptors (HER2)^5^. However, due to the high degree of heterogeneity of breast tumors^6^, relying solely on the clinical characteristics and pathological diagnosis of patients to assess prognosis and formulate treatment plans often results in clinical drugs not being 100% effective, and the clinical results vary greatly^7^. In addition, the existing clinical markers still have certain limitations. Therefore, there is an urgent need to find new molecular diagnostic markers to assess disease progression and the overall prognosis of patients and as potential therapeutic targets.

Long noncoding RNAs (lncRNA) are ncRNAs with a length greater than 200 bp^8^. They can participate in many biological processes by binding with cellular nucleic acids, proteins, and other macromolecules^9^. The Human Genome Project found that only 1.5% of genes are protein-coding genes; most genes are not translated into proteins and are called noncoding genes (ncRNA). LncRNA can be classified into Antisense lncRNAs, Intronic transcript, Large intergenic noncoding RNA, Promoter-associated lncRNA, and UTR-associated lncRNA, according to its sequence characteristics^9^. Due to the different classifications and localizations of lncRNAs, they perform different functions including epigenetic regulation, transcriptional regulation, and post-transcriptional regulation^10,11^. Studies in different cancers have confirmed that lncRNAs play a key role in the occurrence and development of tumors and affect all aspects of cell homeostasis, including cell proliferation, adhesion, migration and apoptosis^12–15^. The same situation happened in breast cancer, Lu G found that, LINC00511 contributes to tumourigenesis and stemness by inducing the miR-185-3p/E2F1/Nanog axis^16^. Xiu reported that, LINC02273 drove breast cancer metastasis by epigenetically increasing AGR2 transcription^17^. Zhang et al. confirmed that. LncRNA DSCAM-AS1 interacted with YBX1 to promote cancer progression by forming a positive feedback loop that activates FOXA1 transcription network^18^. Therefore, searching for key lncRNAs could become a new trend for predicting cancer patient prognosis and identifying potential targets for cancer treatments.

In this study, we conducted a comprehensive study of lncRNA expression profiles in 1048 BRCA samples from The Cancer Genome Atlas (TCGA) datasets. A 6-lncRNA model related to the overall survival (OS) of patients was determined, and a concise nomogram based on the model was developed to improve the prognosis prediction of BRCA patients, which is expected to become a useful diagnostic tool for BRCA patients in the future.

## Results

### Screening of DELs related to OS from BRCA patients

**Figure 1:**
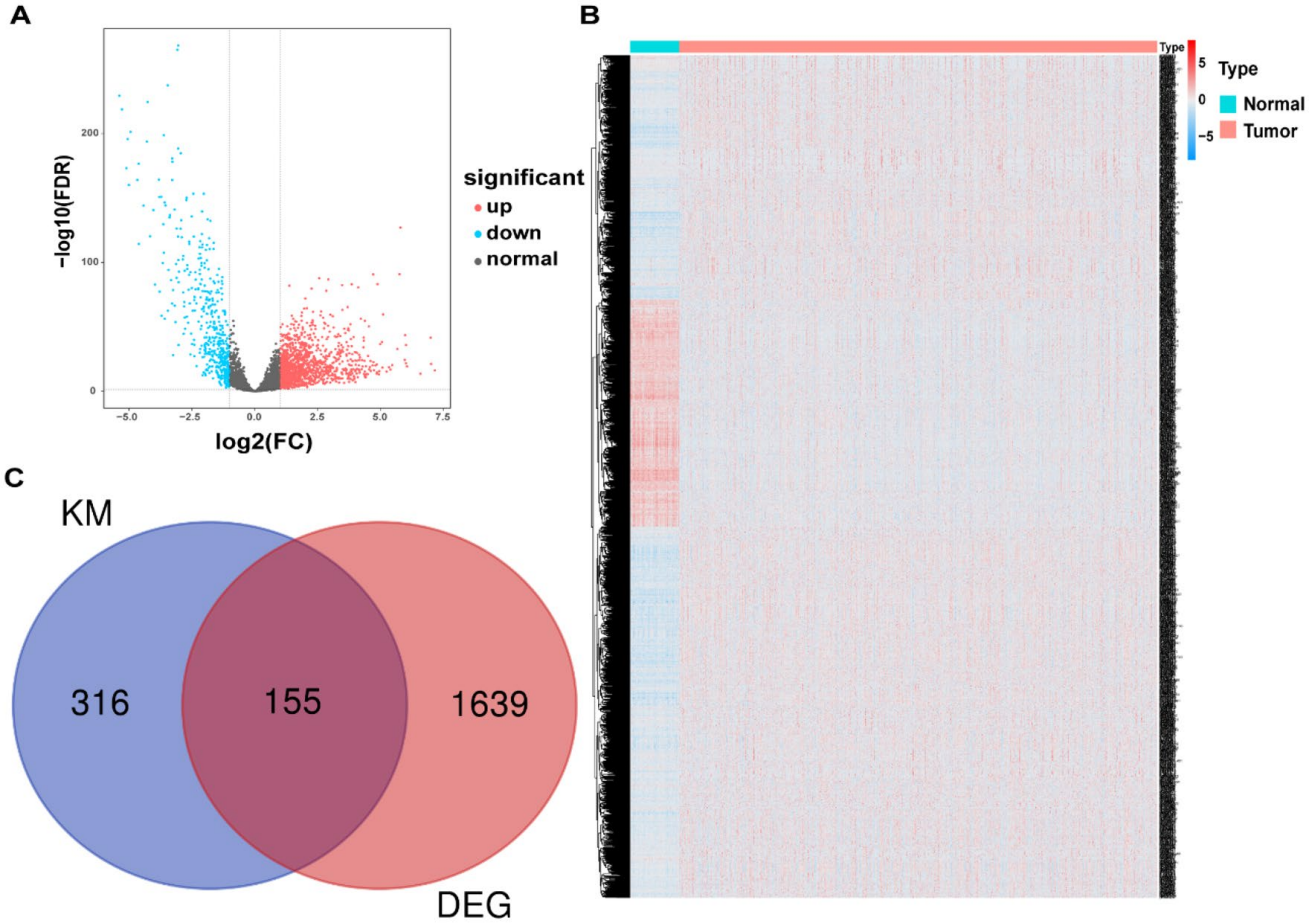
Identify differential lncRNAs (DELs) associated with OS. (**A**) Volcano map of DELs between BRCA tissue and normal breast tissue; (**B**) Heat map of DELs between BRCA tissue and normal breast tissue; (**C**) Venn diagram of OS-related lncRNAs and DELs.

A total of 5673 differentially expressed lncRNAs were obtained from normal and tumor tissues, of which 1794 lncRNAs showed significant differences between the two groups (|logFC|> 1, FDR < 0.05). A total of 1312 lncRNAs were upregulated, while 482 were downregulated, which were visualized in the volcano map and heatmap (Fig. 1A, B). Subsequently, we used the Kaplan–Meier method to screen OS-related lncRNAs among DELs (P < 0.05) and obtained a total of 471 lncRNAs, including 155 significantly different lncRNAs (Fig. 1C). These 155 OS-related DELs served as candidate genes for subsequent analysis.

### Construction and verification of the 6-lncRNA survival prediction model

**Figure 2:**
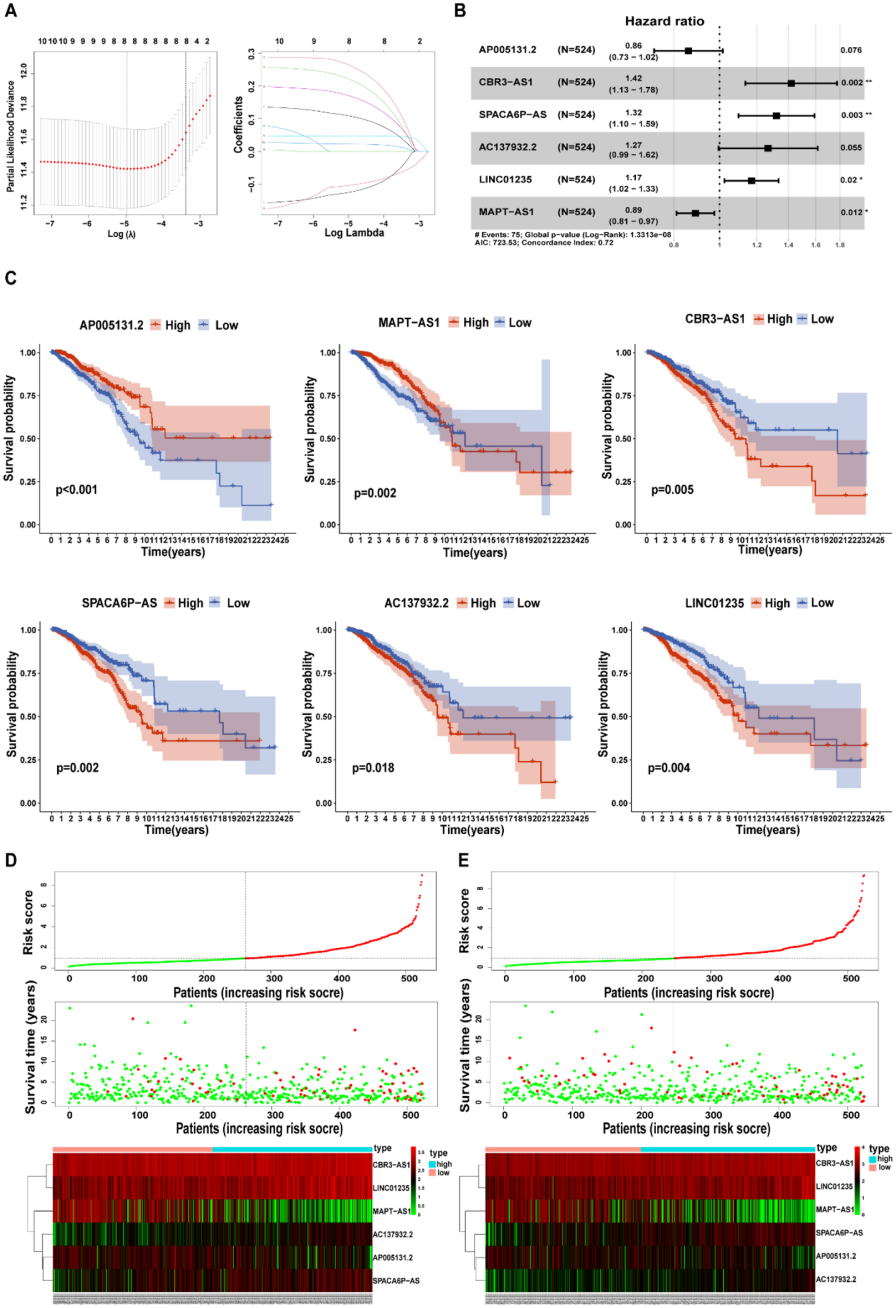
Construction and verification of 6-lncRNA survival prediction model. (**A**) Lasso cox regression analysis to screen OS-related DELs; (**B**) Multivariate Cox regression analysis to build a forest diagram of the risk model; (**C**) Kaplan–Meier to draw the survival curve of each lncRNA in the risk model; (**D**, **E**) the correlation between the risk model and OS in the training group (**D**) and the validation group (**E**). Risk curve (top), Survival status graph (middle), and Risk heatmap (bottom).

A total of 1048 samples were randomly divided into a training set and validation set at a ratio of 1:1. The specific clinical information is shown in Table S1. We used univariate Cox proportional hazard regression analysis on the data in the training set to obtain 10 OS-related lncRNAs (P < 0.01, Table S2), which were further screened by Lasso Cox regression analysis (Fig. 2A). Finally, through multivariate Cox regression analysis, six lncRNAs were selected to construct a risk prediction model (Fig. 2B), and the survival curve of each lncRNA in the model is drawn in Fig. 2C. In the 6-lncRNA model, CBR3-AS1, SPACA6P-AS, AC137932.2 and LINC01235 were considered high-risk lncRNAs, as the expression levels of these lncRNAs were negatively correlated with OS time. The remaining two lncRNAs, AP005131.2 and MAPT-AS1, were low-risk lncRNAs, and their higher expression levels were associated with a longer survival time.

We then created a risk score formula based on the expression of each lncRNA in the model:

$$\begin{aligned} {\text{Riskscore}} & = \left( { - 0.{15} \times {\text{AP}}00{5131}.{2}} \right) + \left( { - 0.{12} \times {\text{MAPT}} - {\text{AS1}}} \right) + \left( {0.{35} \times {\text{CBR3}} - {\text{AS1}}} \right) \\ & \quad + \left( {0.{28} \times {\text{SPACA6P}} - {\text{AS}}} \right) + \left( {0.{24} \times {\text{AC137932}}.{2}} \right) + \left( {0.{16} \times {\text{LINC}}0{1235}} \right). \\ \end{aligned}$$

**Figure 3:**
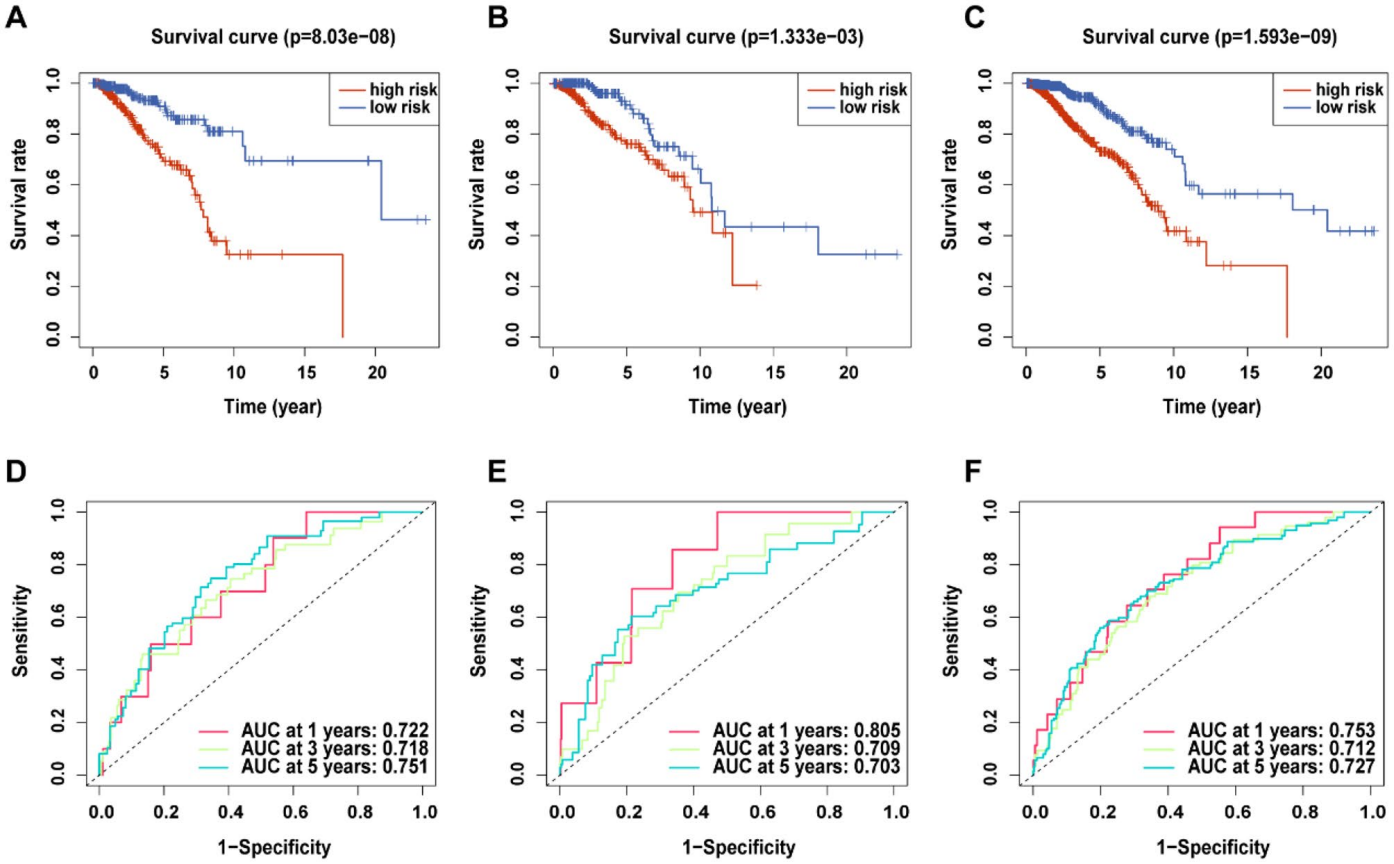
Kaplan–Meier curve and ROC curve show the prediction characteristics and performance of the 6-lncRNA model. (**A**–**C**) Kaplan–Meier to draw the survival curve of riskScore in train set, test set and all set. (D-E) the ROC curves at 1, 3, and 5 years in train set, test set and all set.

Subsequently, we calculated the risk score separately for each sample among the training set, validation set and total set. In each data set, the patients were divided into high-risk groups and low-risk groups based on the median score. Based on the risk score distribution of the high- and low-risk groups in the training set, the OS status and 6-lncRNA model expression profile are shown in Fig. 2D. We found that the OS-time and survival probability of BRCA patients in the high-risk group were lower than those in the low-risk group. It was also observed in the risk heatmap whereby the risk score of patients in the high-risk group was proportional to the expression of high-risk lncRNAs and inversely proportional to the expression of low-risk lncRNAs. Similar results were found in the validation set and the total set (Fig. 2E, Figure S1). Figure 3A shows the Kaplan–Meier curve of the high-risk and low-risk groups in the training set. The OS of the high-risk group was significantly lower than that of the low-risk group (p = 8.03 × e−8). The same results were obtained in both the validation set (Fig. 3B; p = 1.333 × e−3) and the total set (Fig. 3C; p = 1.593 × e−9). In addition, we evaluated the credibility of the 6-lncRNA model by constructing ROC curves at 1, 3, and 5 years. The results showed that in the training set, the AUC values for 1- 3-, and 5-year survival were 0.722, 0.718, and 0.751 (Fig. 3D), respectively, while these values in the verification set were 0.805, 0.709, and 0.703, respectively (Fig. 3E). The AUC values at 1, 3, and 5 years in the total set were 0.753, 0.712, and 0.727, respectively (Fig. 3F). These results indicated that the model has a good predictive ability for survival rates of different lengths.

### The 6-lncRNA model can be used as a clinically independent prognostic factor

**Figure 4:**
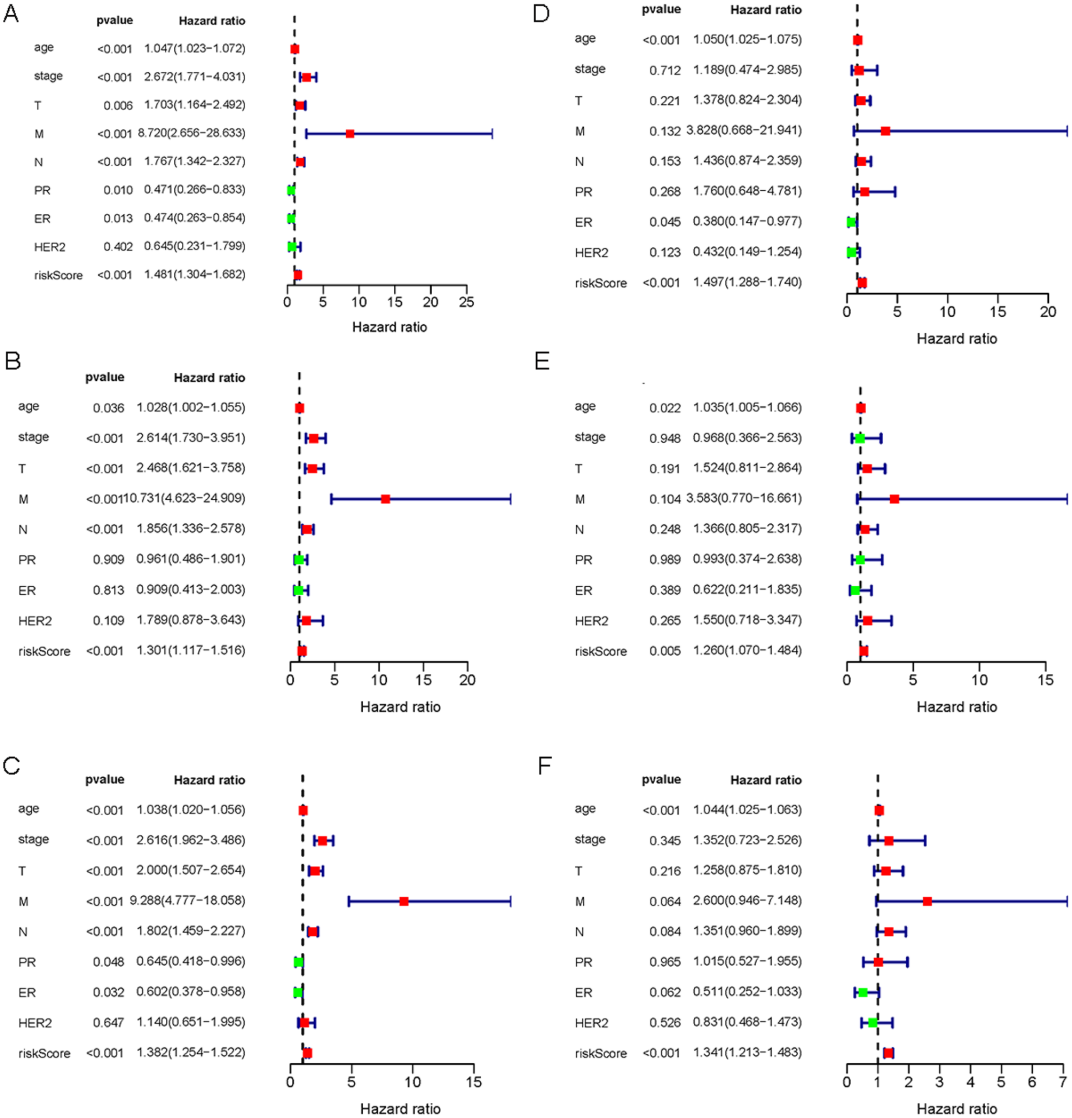
Single-factor and multi-factors Cox regression analysis of OS-related clinical factors. (**A**–**C**) Single-factor Cox regression analysis of OS-related clinical factors in training set (**A**), validation set (**B**) and total set (**C**). (**D**–**F**) Multivariate Cox regression analysis of OS-related clinical factors in training set (**D**), validation set (**E**) and total set (**F**).

To test whether the 6-lncRNA model as a prognostic factor is affected by other clinical risk factors, we first used univariate Cox regression to evaluate the prognostic performance of different clinical features. The results showed that age, disease stage, TNM stage, PR, ER status, and risk model were predictors of OS in the training set (Fig. 4A). In addition, patient age, disease stage, TNM stage, and risk model could also be used as predictors of OS in the validation set and the total set (Fig. 4B, C). Further multivariate Cox regression analysis found that patient age, ER status, and risk model could independently predict the prognosis of BRCA patients in the training set (HR = 1.050, P < 0.001; HR = 0.380, P = 0.045; HR = 1.497, P < 0.001, Fig. 4D). The validation set and total set results show that patient age and the 6-lncRNA risk model are independent prognostic factors for BRCA patients. (HR = 1.035, P < 0.022; HR = 1.260, P < 0.001; HR = 1.044, P < 0.001; HR = 1.341, P < 0.001, Fig. 4E, F).

### The 6-lncRNA model is closely related to the clinicopathological characteristics of BRCA

In order to study the correlation between riskscores and clinical characteristics, we further analyzed the high-risk and low-risk groups and clinical characteristics including age, tumor subtypes, ER, PR, HER2 expression levels, stage status and TNM status. As shown in Table 1, there is a strong correlation between the six lncRNA risk models and clinicopathological characteristics (p < 0.05). The high-risk group is associated with a triple-negative breast cancer (TNBC) subtype with a worse prognosis. In terms of hormone receptor and HER2 expression, the two groups also showed different states. In addition, the risk score in the late stage (stage III–IV) is often higher than the risk score in the early stage (stage I–II). These results indicate that the risk score may be closely related to the worse pathological subtypes of BRCA and tumor progression.

**Table 1:** The association between the clinicopathological characteristics and risk score in BRCA.

	RISK		总计	Pearson X2	P-value
	High	Low			
**Age**					
< 65	381	352	733	0.292	0.589
≥ 65	158	157	315		
All	539	509	1048		
**TNBC**					
+	83	29	112	28.795	**8.05E-08**
−	388	436	824		
All	471	465	936		
**ER**					
+	345	425	770	58.514	**2.02E-14**
−	171	62	233		
All	516	487	1003		
**PR**					
+	282	389	671	76.424	**2.29E-18**
−	235	94	329		
All	517	483	1000		
**HER2**					
+	99	54	153	9.73	**0.001813**
−	273	268	541		
All	372	322	694		
**Stage**					
1–2	382	391	773	5.244	**0.022**
3–4	146	107	253		
All	528	498	1026		
**T**					
1–2	455	425	880	0.035	0.851
3–4	84	81	165		
All	539	506	1045		
**N**					
0	243	251	494	1.705	0.192
1–3	286	251	537		
All	529	502	1031		
**M**					
0	455	418	873	2.507	0.113
1	14	6	20		
All	469	424	893		

### The 6-lncRNA model has good predictive ability in molecular subtypes of breast cancer

In 2000, breast cancer was divided into five subtypes through molecular classification for the first time, named as luminal A, luminal B, HER2/ERB2 enriched, normal-like and basal-like^19^. Each subtype has different clinical and biological behaviors, resulting in different clinical prognosis of patients^19^. We download transcriptome and clinical classification data of different subtypes from TCIA database (https://tcia.at/). We found that the 6-lncRNA modelstill has a good predictive ability for different molecular subtypes of breast cancer. In addition to normal-like breast cancer, patients in the high-risk group of the other four subtypes all have worse OS (Figure S3A-E). And in the ROC curve evaluation of its prediction performance, it is found that each subtype has better prediction accuracy in 1, 3 and 5 years. The AUC values at 1, 3, and 5 years in LumA set were 0.602, 0.734 and 0.669 (Figure S3F), in LumB set were 0.701, 0.772 and 0.754 (Figure S3G), in HER2 set were 0.774, 0.633, and 0.675 (Figure S3H), in basal set were 0.648, 0.614 and 0.733 (Figure S3I). These results indicate that the model can also be used to predict the survival rate of patients with different subtypes of breast cancer.

### Construction and verification of the prognostic nomogram based on the 6-lncRNA model

**Figure 5:**
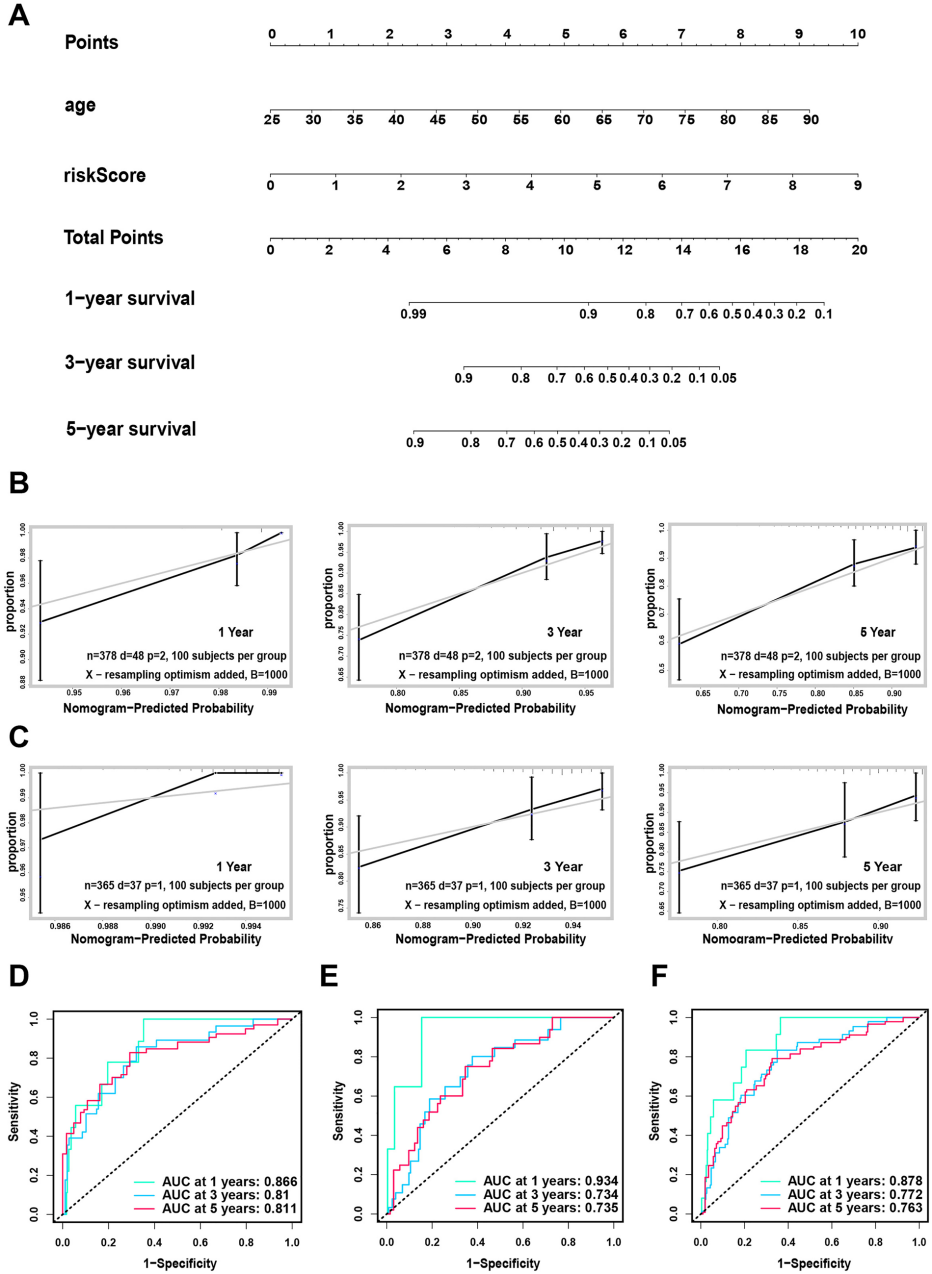
Construction and verification of prognostic nomogram based on 6-lncRNA model. (**A**) The construction of a prognostic nomogram based on the 6-lncRNA model and age; (**B**, **C**) The test set (**B**) and the validation set (**C**) are based on the calibration curve of the nomogram 1, 3, and 5-year predictive ability; (**D**–**F**) The nomograms predict the ROC curve of OS over time in the training set (**D**), the validation set (**E**) and the entire data set (**F**).

Based on the independent prognostic analysis, we found that the age and risk models were shown to be independent prognostic factors in the three data sets. Therefore, we constructed a prognostic nomogram based on these two factors to predict the 1-year, 3-year, and 5-year survival rates of BRCA patients (Fig. 5A). As shown in the nomogram, the 6-lncRNA model contributed the most to the 1-, 3-, and 5-year survival rates, followed by patient age. In addition, we used the consistency index test (C-index) and the calibration chart to evaluate the discrimination ability and calibration ability of the nomogram. Through 1000 resamplings, the internal training set, the validation set and the total set were verified. The results showed that the C index of the training set was 0.790 (95% confidence interval 0.725–0.855), the C index of the verification set was 0.745 (95% confidence interval 0.658–0.832), and the C index of the total set was 0.770 (95% confidence interval 0.718–0.822). Meanwhile, the 1-, 3-, and 5-year prognostic survival rates of BRCA patients predicted by the nomogram were in good agreement with the actual observations in each data set (Fig. 5B, C, Figure S2), indicating that our nomogram is reliable. In addition, we evaluated the prognostic performance through a time-dependent ROC curve. The results showed that the AUC values in the training set for 1, 3, and 5 years were 0.866, 0.81, and 0.811, respectively (Fig. 5D); the predicted AUCs for 1, 3, and 5 years in the validation set were 0.934, 0.734, and 0.735, respectively (Fig. 5E); and the predicted AUC values for 1, 3, and 5 years in the total cohort were 0.878, 0.772, and 0.763, respectively (Fig. 5F).

**Figure 6:**
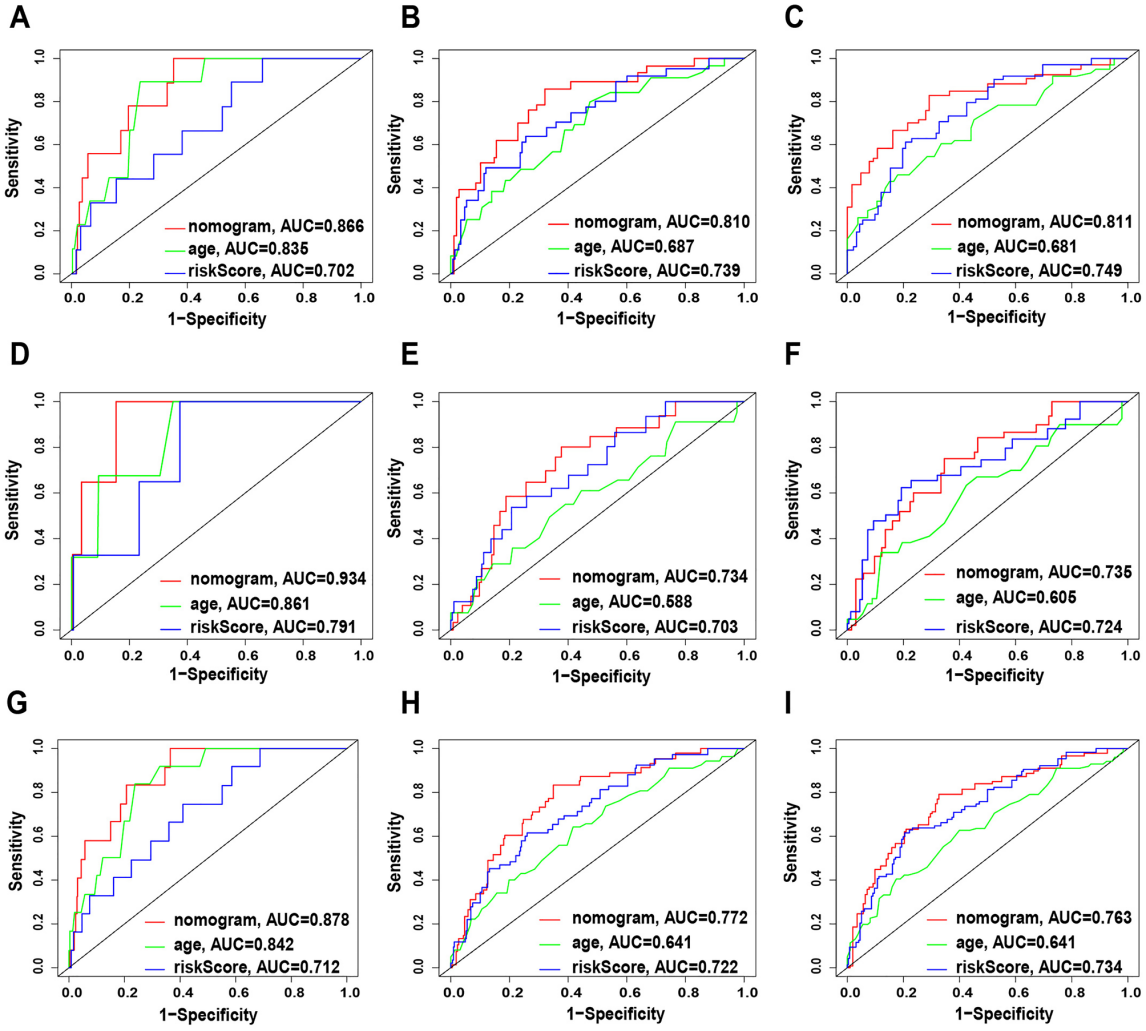
Compare the predictive accuracy and discriminative ability of the nomogram, age and riskScore. (**A**) The ROC curves at 1, 3, and 5 years according to the nomogram, age and riskScore in train set. (**B**) the ROC curves at 1, 3, and 5 years according to the nomogram, age and riskScore in test set. (**C**) The ROC curves at 1, 3, and 5 years according to the nomogram, age and riskScore in all set.

### The nomogram is more reliable than the predictive performance of a single independent prognostic factor

We used a time-dependent ROC curve to compare the prediction sensitivity and specificity between the nomogram and a single independent prognostic factor. The results showed that in the training set, the 6-lncRNA prediction model has a better predictive ability for 3-year and 5-year survival (AUC3 = 0.739; AUC5 = 0.749) than age(AUC3 = 0.687; AUC5 = 0.681), but in terms of 1-year predictive power, age (AUC1 = 0.835) has an advantage over the risk model (AUC1 = 0.702). However, the prediction performance of the nomogram based on 6 lncRNAs was consistently better than that of the risk model or age alone at 1, 3 and 5 years (AUC1 = 0.866; AUC3 = 0.810; AUC5 = 0.811, Fig. 6A). The same conclusion can be drawn in the verification set and the total set (Fig. 6B, C). These results indicated that the nomogram in combination with various clinically independent prognostic factors was more reliable than a single clinical factor.

### Identification of the biological pathways and processes related to the 6-lncRNA model

**Figure 7:**
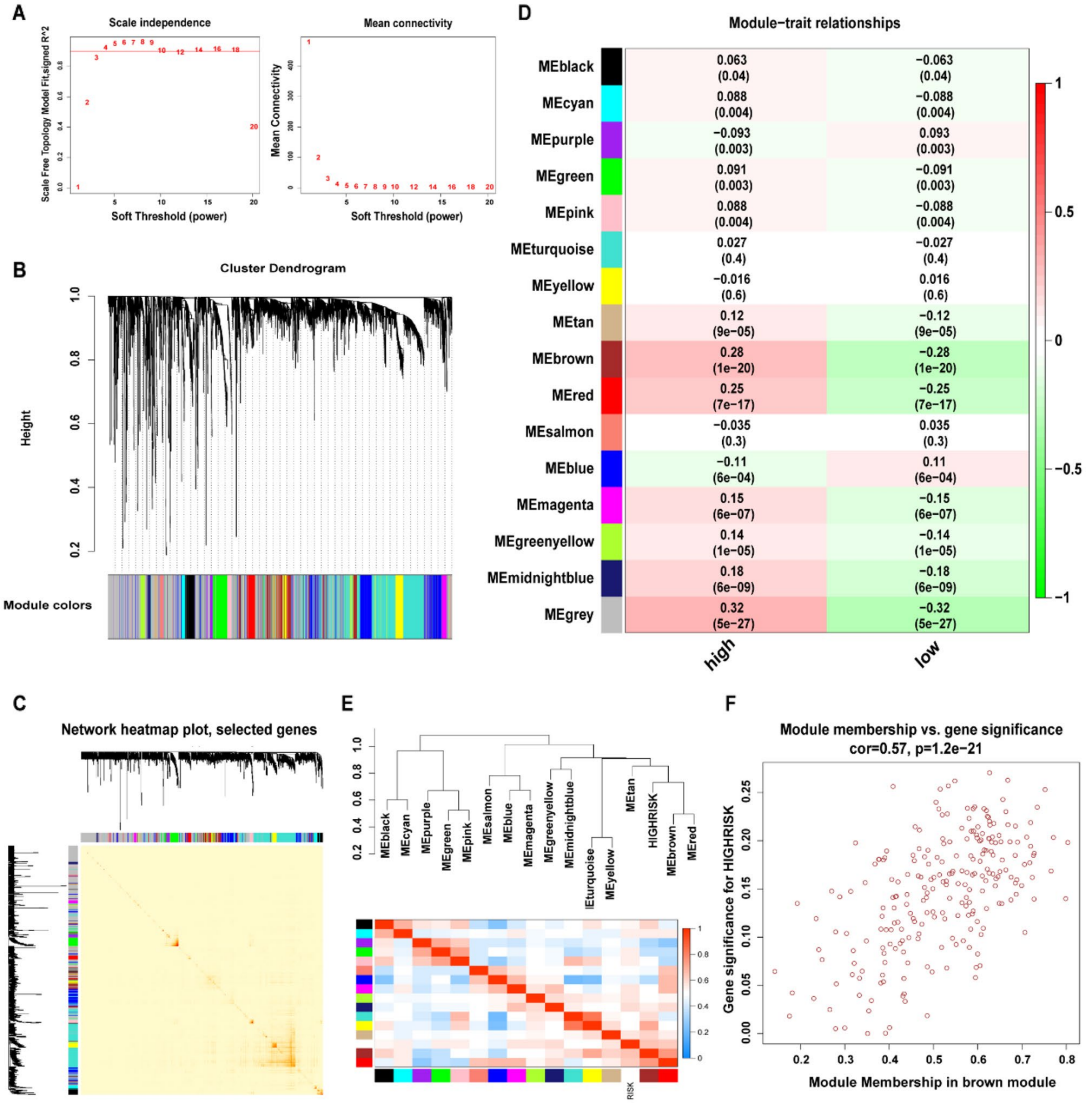
WGCNA analysis. (**A**) Analyze the fitting index of the scale-free topology model with soft threshold (β) and its average connectivity. (**B**) Clustering similar gene dendrograms based on topological overlap and assigned module colors. (**C**) The TOMplot shows the relationship between genes. Light colors indicate lower overlap and dark colors indicate higher overlap. The darker color blocks along the diagonal indicate the co-expression modules. (**D**) Diagram of the correlation between the module and the risk model. Each cell contains the corresponding correlation and P value. (**E**) Dendrogram and heatmap of gene modules. The upper tree diagram shows that the brown module is highly correlated with the risk score. (**F**) Scatter plot of the importance of the relationship between the brown module and the genes in the module.

To explore the potential functions and mechanisms of the components of the 6-lncRNA model, we used the WGCNA method to cluster mRNAs that are highly related to the risk model, which is a common method to find cooperatively expressed gene modules. The soft threshold value was set to 4, creating a scale-free system (Fig. 7A). Modules were generated through dynamic tree cutting. After merging highly similar modules, we generated a total of 16 modules. The "gray" modules contain unexpressed genes and were not used for further analysis (Fig. 7B, C). Additionally, we calculated the correlation between each module and the risk model. The results showed that the brown module had the highest correlation with the risk model (cor = 0.28, P = 1*E−20, Fig. 7D, E), and the genes contained in this module were also highly correlated with the risk model (cor = 0.57, P = 1.2*E−21, Fig. 7F), suggesting that the genes in this module may interact with the 6-lncRNA model; therefore, we selected the genes in this module for subsequent functional and mechanistic analysis.

**Figure 8:**
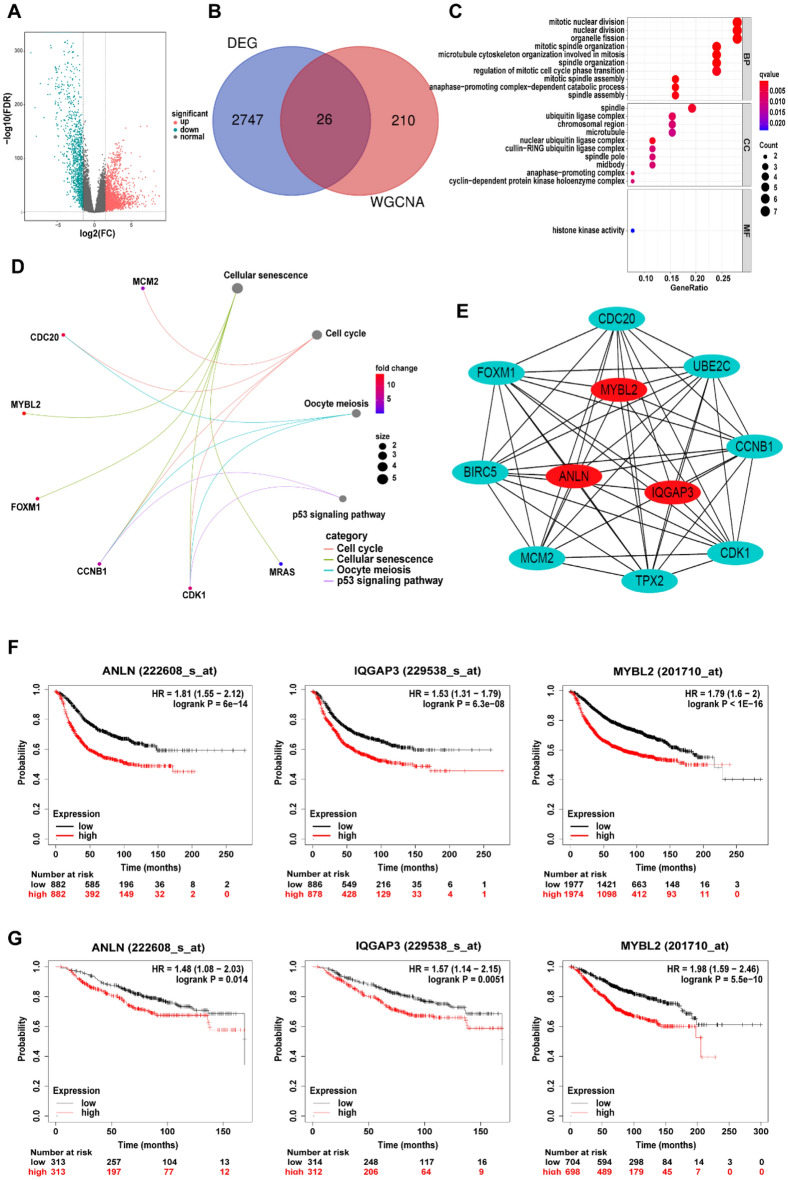
The biological pathways and processes related to the 6-lncRNA model. (**A**) DEMs volcano map between BRCA tissue and normal breast tissue; (**B**) Venn diagram of the brown module genes and DEMs; (**C**) GO enrichment analysis. The biological processes, cellular components and molecular functions of genes are respectively shown. (**D**) Kyoto Encyclopedia of Genes and Genomes (KEGG) pathway analysis of gene-related signal transduction pathways. (**E**) Protein interaction network analysis (PPI): use STRING online tool to construct PPI network; "Cytoscape" "cytoHubba" software package The "ClusteringCoefficient" algorithm screens the first three core genes; (**F**) the recurrence-free survival curve related to the expression of the three core genes; (**G**) the overall survival curve related to the expression of the three core genes.

We obtained a total of 2773 differentially expressed mRNAs (DEMs) between tumor and normal tissue (logFC > 1.5, FDR < 0.05), as demonstrated in a volcano map (Fig. 8A). Then, we intersected the DEMs with the genes in the brown module, where we obtained 26 DEMs that were highly correlated with the 6-lncRNA model (Fig. 8B). These 26 mRNAs were analyzed by GO and KEGG. The results of GO enrichment analysis showed that these DEMs were mainly related to mitosis and proliferation (Fig. 8C). The KEGG enrichment analysis indicated that they were mainly enriched in cellular senescence, the cell cycle, oocyte meiosis, and p53 signaling pathways (Fig. 8D). The activation of these pathways may be able to promote the proliferation and development of tumor cells, leading to a higher risk of death in patients with high risk scores. In addition, we performed PPI analysis on these 26 mRNAs and screened 3 core mRNAs, namely, MYBL2, ANLN and IQGAP3 (Fig. 8E). The online tool Kaplan–Meier Plotter was used to analyze the RFS and OS associated with these three core genes. The results showed that the high expression of these three core genes led to worse RFS and OS in BRCA patients (Fig. 8F, 8G). In summary, the 6-lncRNA model could regulate the occurrence and development of tumors by affecting the mitosis and proliferation of tumor cells and ultimately lead to poor survival prognosis for high-risk patients.

### Immune infiltration characteristics of 6-lncRNA model

**Figure 9:**
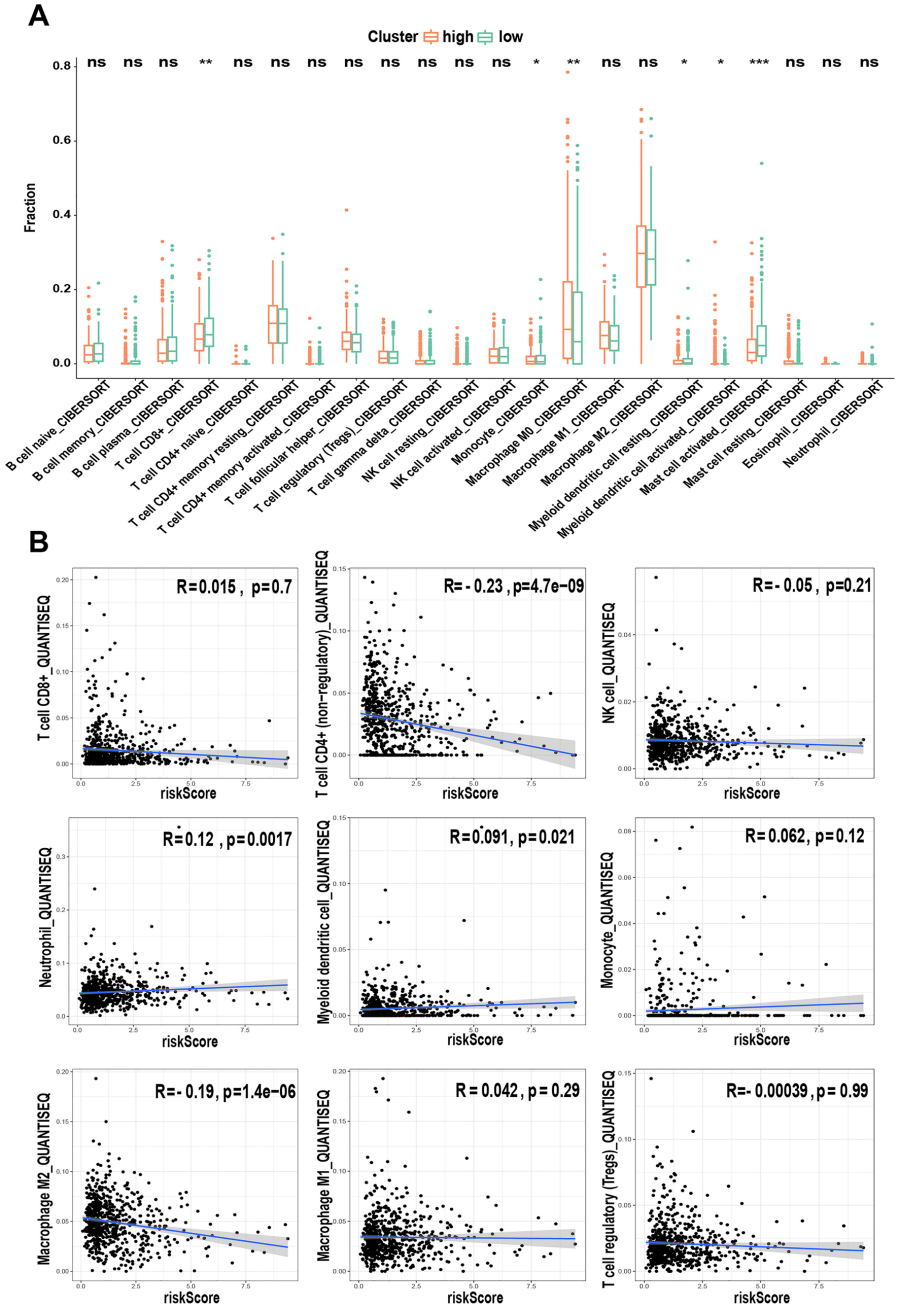
Immune infiltration differences and correlation analysis between high and low risk groups. (**A**) Box plot shows the expression differences between the two groups of 22 immune cells. (**B)** Spearman's correlation coefficient calculates the correlation between immune cells and risk scores. The P value is shown as: ns, not significant; *P < 0.05; **P < 0.01; ***P < 0.001.

In order to further explore the relationship between riskscore and immune cells, we obtained quantitative results of immune cell infiltration from TIME2.0. The results showed that among 22 kinds of immune cells, 6 kinds of immune cells have differential expression in different risk groups. Compared with the high-risk group, the low-risk group has higher CD8+T cells, macrophages and other cells that are beneficial to killing tumor cells, while the high-risk group has a higher level of M0 macrophages. (Fig. 9A) To further understand the relationship between immune cells and risk models, we use QUANTISEQ algorithm to calculate the correlation between immune cell infiltration results and risk scores. The QUANTISEQ algorithm is a deconvolution algorithm specially developed for RNA-seq data. It has high deconvolution performance on tumor data from different cancer types^20^. The results showed that the risk score was significantly negatively correlated with CD4+T cells (R = -0.23; P = 4.7e−09), and with neutrophils (R = 0.12; P = 1.7e−03) and myeloid dendrites Shape cells (R = 0.091; P = 2.1e−02) showed a weaker positive correlation (Fig. 9B).

## Discussion

Breast invasive carcinoma is the most common cancer in women^3^. According to global cancer statistics, there were more than 1.7 million new diagnoses of breast cancer in 2012, accounting for 25% of all cancers^21^. In addition, breast cancer incidence increases rapidly with age^22^. In some developed countries, up to 6% of women are diagnosed with invasive breast cancer before the age of 75^3^. The diagnosis and treatment of traditional BRCA mainly depend on the TNM stage, histological grade, and hormone receptor status of the disease^5^. Patients with early breast cancer respond well to traditional diagnosis and treatment. However, due to the high degree of heterogeneity of breast cancer^6^, it is not feasible for every patient to rely only on the currently available treatment options and prognostic evaluation methods^7^. Therefore, the study of novel biomarkers and models is essential to improve BRCA prognosis prediction and therapeutic target discovery, as well as treatment options.

More and more lncRNAs with different functions have been found in the study of breast cancer. Li et al. found that LncRNA ANCR could reduce the invasion and metastasis of breast cancer by mediating the degradation of EZH2^23^. Zhao W reported that LncRNA HOTAIR affected the growth, migration, invasion and apoptosis of breast cancer cells through miR-20a-5p/HMGA2 axis^24^. Kong et al. found that lncRNA-CDC6 can promote the proliferation and metastasis of breast cancer cells through the mechanism of ceRNA^25^. Among these dysregulated lncRNAs, some have the potential to be critical biomarkers for early detection and prognosis prediction^26,27^. To date, several lncRNA prognostic models have been proven to enhance the prognostic performance of different cancers^28,29^.

In this study, we downloaded expression data and clinical information for BRCA samples from the TCGA database. Through bioinformatics and statistical analysis, we constructed a 6-lncRNA risk prediction model based on the training set and proved the reliability of the model's predictive potential in the verification set and the total set. Independent prognostic analysis found that the 6-lncRNA model was not affected by other clinical factors, such as patient age, disease stage, TNM stage or hormone receptor status. Thus, the 6-lncRNA model could be used as an independent prognostic factor for BRCA.

In our research, the 6 lncRNAs found were CBR3-AS1, SPACA6P-AS, AC137932.2 AP005131.2, MAPT-AS1 and LINC01235. Among the six lncRNAs, five were antisense lncRNAs. The localization of MAPT-AS1 may in cytoskeleton or nucleus, localization of CBR3-AS1 may in extracellular, cytoskeleton or nucleus. The information of 6 lncRNAs were showed in Table 2. Of these 6 lncRNAs, some have been shown to be related to the occurrence and development of tumors in previous studies. CBR3-AS1 (also known as plncRNA1) has been reported as an oncogenic factor in various tumors. It was initially found to be highly expressed in prostate cancer (PCa)^30^ and to interact with androgen receptor to promote PCa cell proliferation, migration and invasion, further leading to a reduced survival rate^31^. This lncRNA was also upregulated in hepatocellular carcinoma, gastric cancer, esophageal squamous cell carcinoma^32^, nonsmall cell lung cancer^33^, colon cancer^34^ and glioma^35^ and was associated with poor prognosis. In Xu's study^36^, CBR3-AS1 was found to be a cancer-promoting factor associated with a worse prognosis of breast cancer. LINC01235 upregulation in gastric cancer can induce EMT^37^. Vishnubalaji^38^ suggested that the upregulation of LINC01235 in breast cancer may represent a more aggressive phenotype. SPACA6P-AS (SP-AS) participates in the formation of the ceRNA network, thereby regulating the establishment and development of liver cancer^39^. MAPT-AS1 has been shown to be closely related to the prognosis of many cancers. The low expression of MAPT-AS1 in clear cell renal cell carcinoma indicates a lower overall survival rate even after nephrectomy^40^. Wang^41^ reported that MAPT-AS1 showed low expression in the high immunity group of glioma cells and predicted worse survival. Another^42^ study confirmed that the expression of MAPT-AS1 is associated with a better prognosis of nontriple-negative breast cancer. These findings are consistent with our research.

**Table 2:** Information of six lncRNAs.

Gene symbol	Ensembl ID	Location (hg38)	Class	Localization
AP005131.2	267366	chr18:13,500,640–13,501,289	Antisense	No data
			C18orf1	
MAPT-AS1	264589	chr17:45,799,389–45,895,680	MAPT antisense RNA 1	Cytoskeleton
				/Nucleus
CBR3-AS1	236830	chr21:36,130,371–36,276,226	CBR3 antisense RNA 1	Extracellular
				/Cytoskeleton
				/Nucleus
SPACA6P-AS	269959	chr19:51,682,683–51,693,456	SPACA6 antisense RNA 1	No data
AC137932.2	261253	chr16:89,321,133–89,325,110	Antisense to ANKRD11	No data
LINC01235	270547	chr9:13,404,750–13,488,226	Long intergenic non-protein coding RNA 1235	No data

The nomogram is a common tool used to predict survival and can integrate various continuous or discontinuous prognostic variables. Scores can be calculated in tumor patients according to different variable parameters; thus, we can assess the corresponding survival probability. It is intuitive and easy to understand, making it easy for clinicians to use^43^. In this study, we integrated the 6-lncRNA model with traditional clinically independent prognostic factors and developed a prognostic nomogram that includes the 6-lncRNA model and patient age to predict prognosis. By comparing the predictive ability of the nomogram with single independent prognostic factors, we showed that a nomogram combining multiple independent prognostic factors could have better prediction accuracy for different survival times than a single factor. The nomogram we constructed can be used as a practical and reliable prognostic tool for BRCA patients.

WGCNA is a common method used to find cooperatively expressed gene modules or to explore the association between gene networks and phenotypes of interest. This study uses this method to screen out the gene modules most closely related to the 6-lncRNA model. Furthermore, the functions and pathways of modular genes were found to be related to cell cycle regulation, cell migration and cell survival. In addition, the roles of the three core genes in the module in regulating tumors have been reported in previous review studies.

MYBL2 is a member of the MYB transcription factor family. It plays an important role in regulating the cell cycle, cell migration and cell survival. It is overexpressed in many solid tumors and associated with poor prognosis^44^. MYBL2 is highly expressed in more aggressive subtypes of breast cancer, such as TNBC. Higher expression levels of MYBL2 promote cell proliferation and lead to genome instability through DNA damage, which results in the expansion of aneuploid cells and promotes epithelial-mesenchymal transition and therapeutic resistance. Targeting this multifunctional protein may be an effective treatment to prevent breast cancer recurrence^45^. ANLN is an actin binding protein that is reported to play a vital role in cell proliferation and migration, especially in cytokinesis^46^. ANLN is highly expressed in a variety of cancers and promotes the development of cancer^47^. Therefore, ANLN may be an effective target for cancer treatment, but the relationship between ANLN and cancer is not fully understood and needs further research^46^. IQGAP3 belongs to the IQGAP family and has been found to be responsible for regulating a variety of cellular processes, including cytokinesis, cell migration, cell proliferation, intracellular signal transduction, vesicle transport, and cytoskeletal dynamics^48^. A number of studies have shown that there is a strong correlation between the expression of IQGAP3 and poor prognosis in various cancers^49–51^. Hu et al.^52^, found that the expression of IQGAP3 is significantly increased in breast cancer tissues and regulates tumor cell proliferation and metastasis. IQGAP3 was inhibited by increasing p53 expression and reducing MMP9, Snail, Twist, CDC42, p-ERK1/2, KIF2C, KIF4A and PCNA, thereby inhibiting the growth, migration and invasion of breast cancer cells^52^. Therefore, IQGAP3 may be a potential therapeutic target in human breast cancer. In our study, the three core genes had a significant impact on the RFS and OS of BRCA patients, which is consistent with previous studies and proves that the 6-lncRNA model we constructed may serve as a useful tool in predicting the occurrence and development of BRCA.

In recent years, immunotherapy has achieved good results in various tumors^53^. It uses the body's own defense system to fight cancer, or uses immune checkpoint inhibitors to eliminate the immune tolerance of tumor cells, so as to achieve the purpose of treatment and has broad application prospects^54^. Our results show that the immune status between the low-risk score group and the high-risk score group is significantly different, and the low-risk group has stronger anti-tumor immunity, showing that patients with different strata have a complex tumor immune environment. It is expected to provide reference for immunotherapy.

Although our 6-lncRNA risk model and nomogram are effective in predicting the prognosis of BRCA patients, the study still has some limitations. First, the TCGA database lacks More parameters that affect patient survival, such as smoking, radiotherapy, targeted therapy, etc., and these parameters will be helpful to construct additional layers of analysis. Second, as this is a retrospective study based on database information, future prospective studies with large sample sizes are needed to verify the reliability of the nomogram's predictive ability. Meanwhile, the specific mechanisms of how these lncRNAs modulate the prognosis of BRCA patients requires further attention.

## Conclusion

In general, lncRNAs can be used as meaningful predictors in a variety of cancers. The 6-lncRNA risk model and nomogram constructed in this study have independent predictive ability, with predictive value better than that of a single factor, and can be used as practical and reliable prognostic tools for BRCA. The nomogram provides additional clinical value for the individualized survival prediction and treatment options of BRCA patients to help individualized management of patients.

## Materials and methods

### Analysis process and data processing

**Figure 10:**
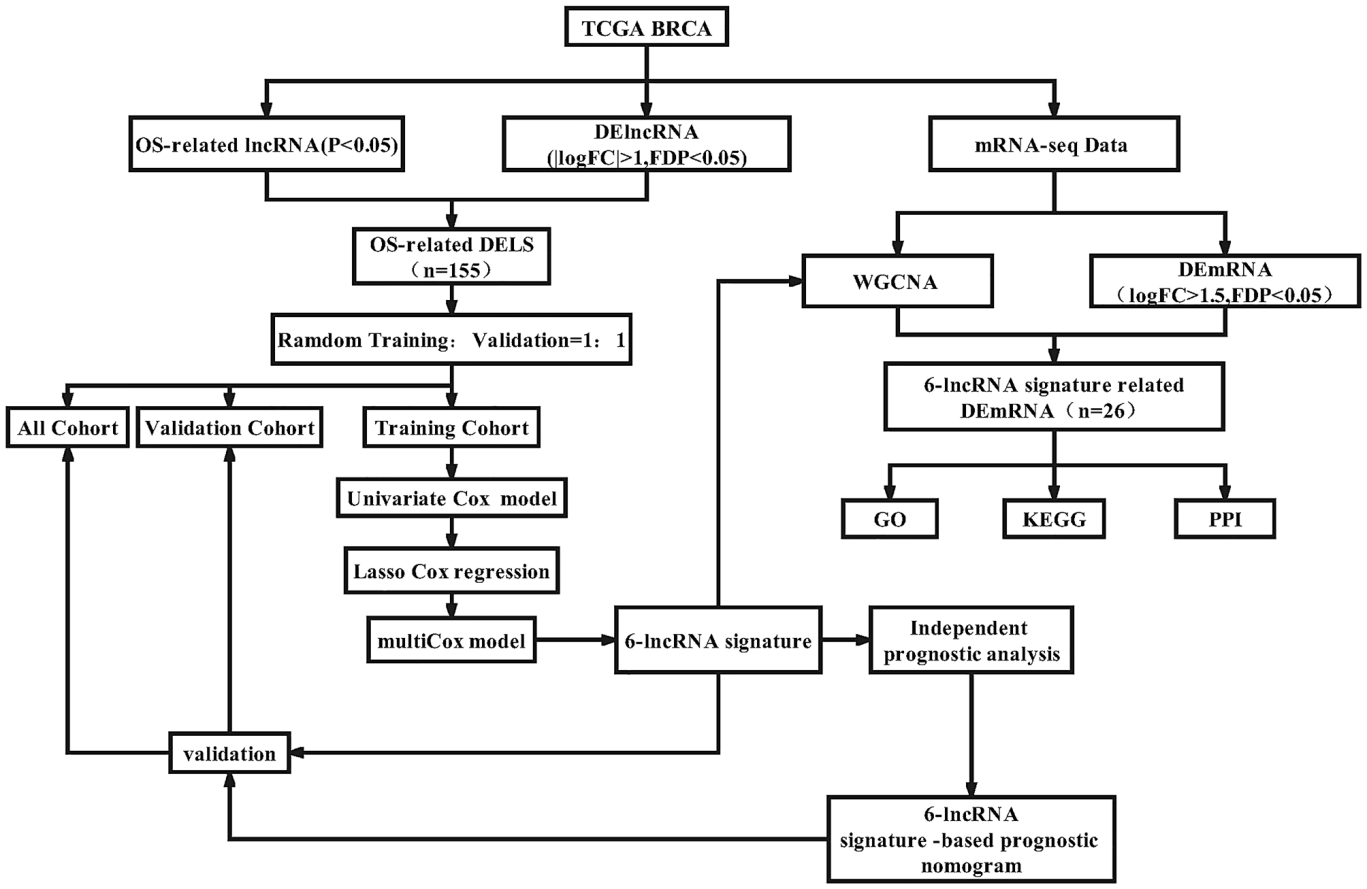
Work flow chart.

The analysis process is shown in Fig. 10. The RNA-seq data and corresponding clinical information of BRCA patients come from The Cancer Genome Atlas (TCGA) datasets (TCGA: https://portal.gdc.cancer.gov/); a total of 1208 samples were obtained, including 112 healthy samples and 1096 BRCA Sample, use Gencode (Gencode v34 GRCh38) GTF file to annotate BRCA data. The data collection and processing of our research complies with TCGA's data policy to protect human subjects.

### RNA-seq difference analysis

We standardize the downloaded RNA-seq data and use the "edgeR" package (3.32.1) for differential expression analysis. The lncRNA screening condition is |logFC|> 1, FDR < 0.05; the mRNA screening condition is |logFC|> 1.5, FDR < 0.05 as the gene with significant difference for subsequent analysis. "pheatmap" package (1.0.12) and " ggplot2" package (3.3.5) are used to draw the heatmap and volcano map.

### Identify OS-related differential lncRNAs in BRCA patients

We obtained a total of 1085 clinical data. Patients with a follow-up time of fewer than 10 days and insufficient survival data were excluded, and 1048 clinical data were retained for subsequent prognosis analysis and model construction. The Kaplan–Meier method was used to screen lncRNAs related to prognosis. Only P < 0.05 was considered as a candidate for lncRNA related to OS. Then, the candidate lncRNA and the DELs obtained in the above steps are intersected to obtain DELs related to OS.

### Construction of the lncRNA prognostic model

We randomly divided 1048 samples into training set and validation set at a ratio of 1:1. In the training set, lncRNAs with a p < 0.01 in the univariate Cox regression analysis were selected for further screening by the least absolute shrinkage and selection operator (LASSO) Cox regression analysis. Finally, the prognostic model of lncRNAs was constructed through a multivariate Cox regression analysis. Using Kaplan–Meier, time-dependent ROC curve evaluates the model's predictive ability on the training set, validation set and the entire data set. The risk curve, Survival status graph, and Risk heatmap were drawn by "pheatmap" package (1.0.12) in R software (4.0.2).

### Development of prognostic nomogram based on lncRNA model

We used univariate and multivariate Cox to analyze the prediction effect of the 6-lncRNA model and traditional clinical risk factors (including age, disease stage, TNM stage, ER, PR and HER2 status). As an independent prognostic clinical factor, the "rms" package (6.2-0) is used to construct a prognostic nomogram. The C index and the calibration curve were used to perform 1000 re-sampling verification to evaluate the prediction accuracy of the nomogram on the training set, the verification set and the entire data set. And through the time-correlation ROC curve to assess and compare the predictive performance of the nomogram itself and compared with other risk factors.

### Weighted gene co-expression network analysis (WGCNA)

In order to find the mRNA modules closely related to the 6-lncRNAs model, we conducted the WGCNA analysis in the R software "WGCNA (1.70-3)" package^55^. We selected the top 25% mRNAs with the largest variance in the samples and removed two outlier samples, the soft threshold is set to 4 to construct a scale-free network graph and a topological matrix (TOM). Using the "dynamic cutting tree" method (cutHeight = 8,000,000, minSize = 10, mergeCutHeight = 0.3), the co-expressed genes are classified into the same module. The correlation between each module and the risk model was calculated. And the module with the maximal associated with lncRNAs model was selected out. The TOMplot and dendrogram and heatmap were drawn by "WGCNA (1.70–3)" package.

### Function and pathway enrichment and protein interaction network analysis

We intersected the genes in the module that are most relevant to the risk model and the DEMs obtained from the above steps, and select key mRNAs for further analysis. Use R "clusterProfiler (3.18.1)", "org.Hs.eg.db (3.12.0)", "enrichplot (1.10.2)" package, set FDR < 0.05 as the filter condition, and perform Gene Ontology (GO) biological processes and Kyoto Encyclopedia of Genes and Genomes (KEGG) Pathways analysis^56–58^. We used the online tool STRING^59^ to construct a PPI network to further understand the protein interactions between genes in the module. The first three core genes were screened out through the Cytoscape: "ClusteringCoefficient" algorithm of "cytoHubba", and the online tool Kaplan-Meier^60^ was used. Plotter analyzes the recurrence-free survival rate (RFS) and OS rate of core genes.

### Tumor immune infiltration analysis

We use the TIME2.0 database^61^ (http://timer.cistrome.org/) to obtain the quantitative results of tumor immune infiltration under six different algorithms. The Kruskal–Wallis test was used to compare the difference between the 22 immune cell infiltrations in the high and low riskscore groups; the Spearman correlation coefficient was used to calculate the correlation between immune cells and the risk score under the QUANTISEQ algorithm.

### Statistically analysis

All statistical analysis was performed using R software (4.0.2) and SPSS (23.0). Use "edgeR" package (3.32.1) for differential expression analysis; "glmnet"(4.1-2) and "survminer (0.4.9)" packages for Cox analysis and lasso regression analysis. Use "timeROC (0.4)" for time-related ROC analysis and area under the curve (AUC) calculation; use "survival (3.2-11)" package for survival analysis. Kruskal–Wallis test was used to assess the significance of differences in immune cell components; Pearson's chi-squared test was used to calculate the correlation between Clinical features and risk cluster. Spearman correlation coefficient was used to calculate the correlation between immune cells and the risk score. P < 0.05 was considered statistically significant (P < 0.05, "*"; P < 0.01,"**"; P < 0.001,"***").

### Ethics approval and consent to participate

Not applicable.

### Consent for publication

Not applicable.

## Supplementary Information

Supplementary Information.

## Data Availability

All data comes from The Cancer Genome Atlas (TCGA) datasets (TCGA: https://portal.gdc.cancer.gov/).
