# Supplementary material for: The nomogram based on the 6-lncRNA model can promote the prognosis prediction of patients with breast invasive carcinoma
Source: Sci Rep. 2021 Oct 21;11:20863. doi: 10.1038/s41598-021-00364-w (PMC8531445; doi:10.1038/s41598-021-00364-w)
Supplement: Supplementary file 1 — Supplementary Information. [file 41598_2021_364_MOESM1_ESM.docx]

| Table S1 |  |  |  |
| --- | --- | --- | --- |
| **Clinical features of BRCA patients in the training and validating groups.** | | | |
|  |  | Train | Test |
| age | >65 | 146 | 149 |
|  | ≤65 | 378 | 375 |
|  |  |  |  |
| stage | I-II | 377 | 396 |
|  | III-IV | 136 | 117 |
|  |  |  |  |
| T | T1-2 | 434 | 446 |
|  | T3-4 | 89 | 76 |
|  |  |  |  |
| N | N0 | 243 | 251 |
|  | N1-3 | 272 | 265 |
|  |  |  |  |
| M | M0 | 442 | 431 |
|  | M1 | 7 | 13 |
|  |  |  |  |
| ER | （+） | 282 | 284 |
|  | （-） | 96 | 81 |
|  |  |  |  |
| PR | （+） | 258 | 241 |
|  | （-） | 120 | 124 |
|  |  |  |  |
| HER2 | （+） | 58 | 78 |
|  | （-） | 320 | 287 |
|  |  |  |  |
| status | live | 449 | 453 |
|  | dead | 75 | 71 |

| Table S2 | |  |  |  |
| --- | --- | --- | --- | --- |
| **Ten lncRNAs significantly associated with the OS derived from the univariable Cox proportional hazards regression analysis.** | | | | |
| id | HR | HR.95L | HR.95H | pvalue |
| AP005131.2 | 7.83E-01 | 6.61E-01 | 9.26E-01 | 4.36E-03 |
| CBR3-AS1 | 1.36E+00 | 1.09E+00 | 1.71E+00 | 7.44E-03 |
| SPACA6P-AS | 1.27E+00 | 1.06E+00 | 1.52E+00 | 8.69E-03 |
| MAPT-IT1 | 8.73E-01 | 8.00E-01 | 9.53E-01 | 2.50E-03 |
| LINC00668 | 1.15E+00 | 1.08E+00 | 1.23E+00 | 4.84E-05 |
| AC137932.2 | 1.42E+00 | 1.12E+00 | 1.79E+00 | 3.25E-03 |
| LINC01235 | 1.21E+00 | 1.08E+00 | 1.36E+00 | 1.38E-03 |
| MAPT-AS1 | 8.50E-01 | 7.82E-01 | 9.23E-01 | 1.23E-04 |
| LINC01016 | 9.10E-01 | 8.50E-01 | 9.74E-01 | 6.89E-03 |
| LINC01456 | 1.14E+00 | 1.04E+00 | 1.25E+00 | 3.51E-03 |


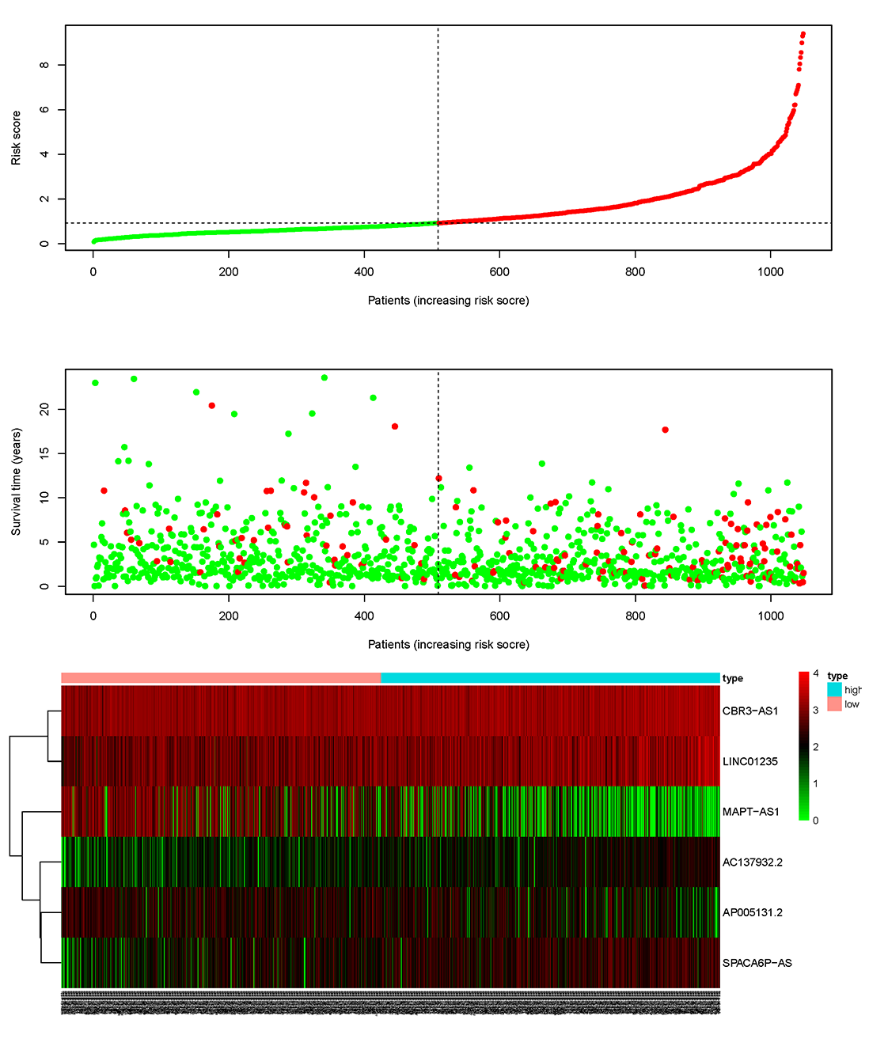


Figure S1: The correlation between the risk model and OS in the total set.


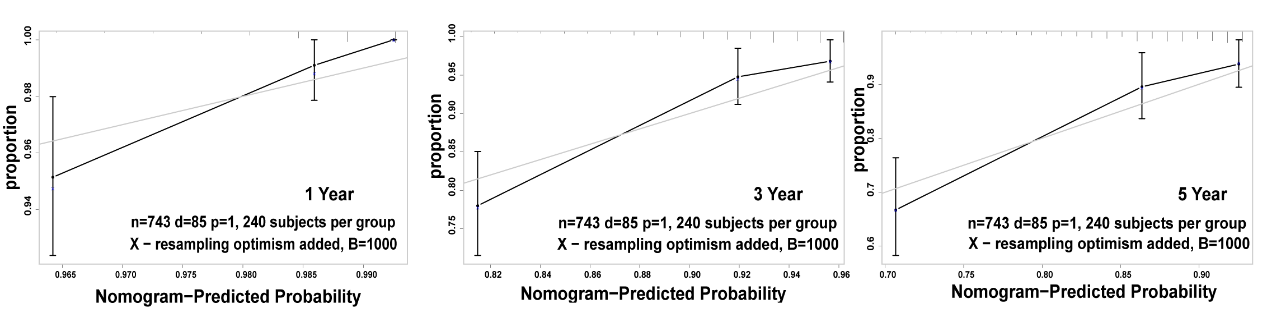


Figure S2: The tatal set are based on the calibration curve of the nomogram 1, 3, and 5-year predictive ability.


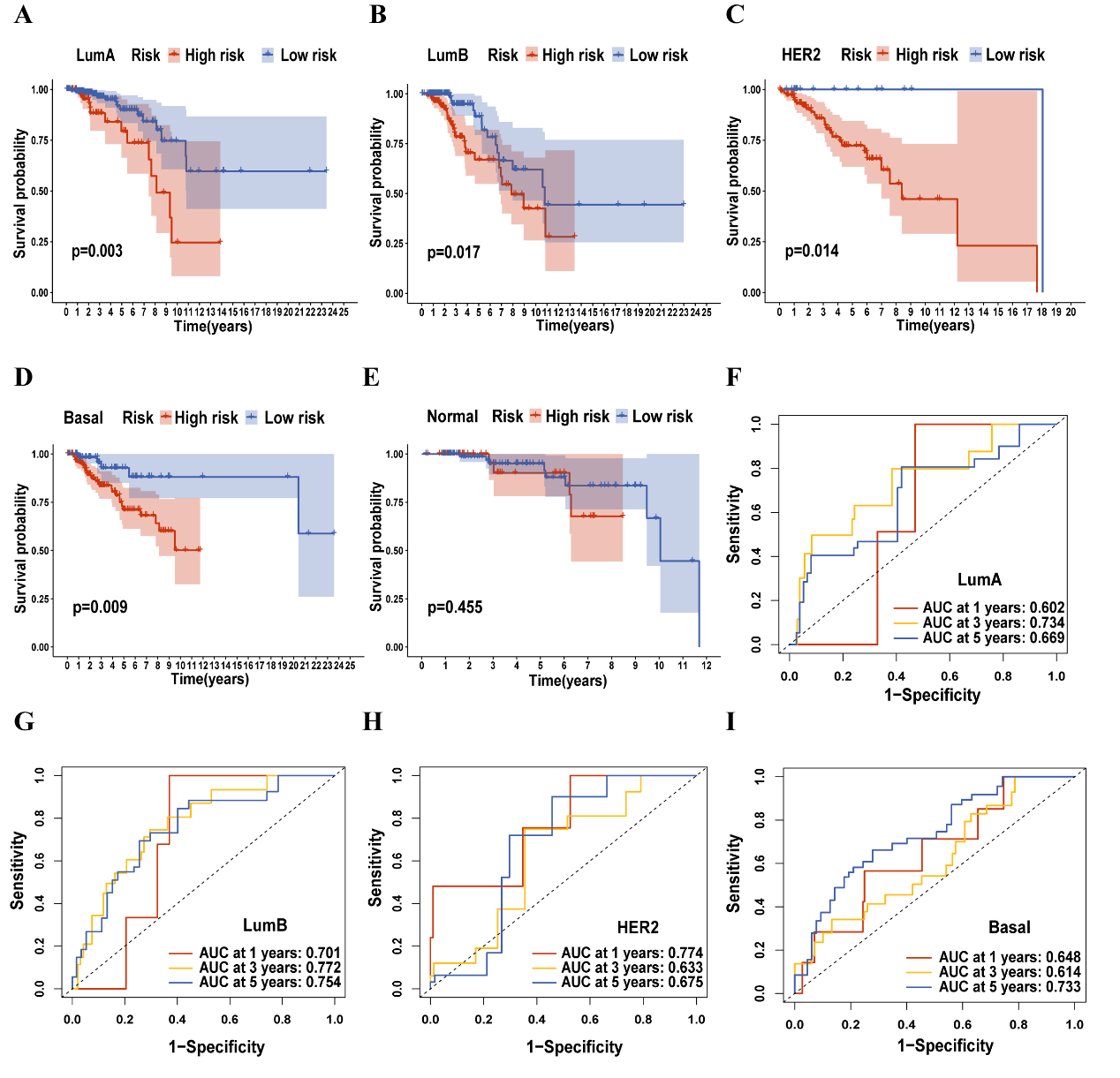


Figure S3: Evaluated the predictive ability and accuracy of the 6-lncRNA model in molecular subtypes of breast cancer. (A-E) Kaplan-Meier to draw the survival curve of riskScore in LumA, LumB, Her2 enriched, basal-like and normal-like subtypes. (F-I) The 6-lncRNA model predict the ROC curve of OS over time in LumA (F), LumB (G), Her2 enriched (H) and basal-like subtypes (I).
